# Axillary nodal metastasis at primary presentation of an oropharyngeal primary carcinoma: a case report and review of the literature

**DOI:** 10.4076/1752-1947-3-7230

**Published:** 2009-08-13

**Authors:** Bruce J Mckenzie, James W Loock

**Affiliations:** 1 Department of Otorhinolaryngology, Faculty of Health Science, University of Stellenbosch, Tygerberg, 7505, South Africa

## Abstract

**Introduction:**

Axillary nodal metastasis is very rare in head and neck squamous cell carcinoma. The few cases reported in the literature all involve patients who have previously undergone either neck dissection alone, or neck dissection and radiotherapy to the neck, and subsequently develop delayed recurrences of disease, with axillary nodal involvement.

**Case presentation:**

We present the case of a 62-year-old man of Cape Malay ethnicity, who presented with an oropharyngeal squamous cell carcinoma, and cervical and axillary nodal metastasis at primary presentation.

**Conclusion:**

Whilst previous reports in the literature suggest routine examination of the axilla is advisable in patients with previously treated neck cancer and recurrence of head and neck cancer, we propose that the axilla should be routinely examined in new cases, particularly when there is involvement of the level 5 nodes.

## Introduction

The management of the neck in squamous cell carcinoma of the head and neck is based on the predictable pattern of lymphatic spread of disease through the cervical lymph nodes. Unpredictable spread rarely occurs. The literature reports few cases of axillary node involvement in head and neck carcinoma, all found in patients who have previously undergone either neck dissection alone, or neck dissection and radiotherapy to the neck.

This case report presents the first reported incidence where the axillary nodes are involved in the primary presentation of a patient presenting with squamous cell carcinoma (SCC) of the upper aerodigestive system.

## Case presentation

A 62-year-old Cape Malay man presented to our department in June 2006 with a 3-month history of an ulcerative lesion on his left tonsil. He had noticed left-sided cervical adenopathy in the preceding 2 months. He first noticed a left axillary mass 6 weeks before presentation.

**Figure 1:**
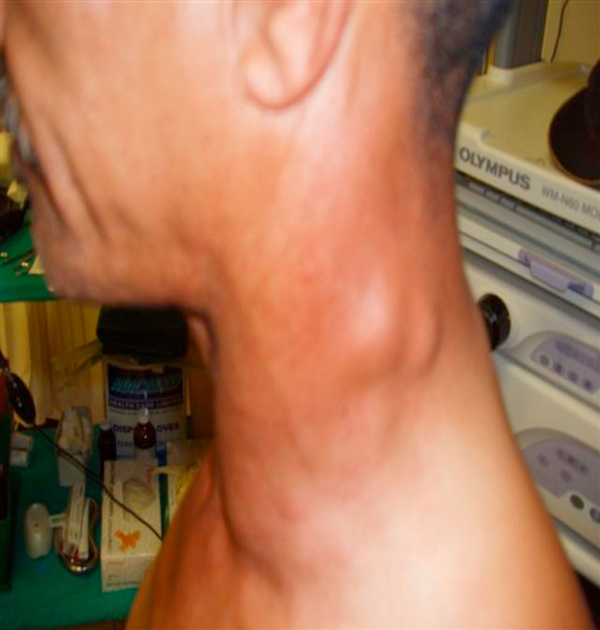
**Level 5 nodes**.

**Figure 2:**
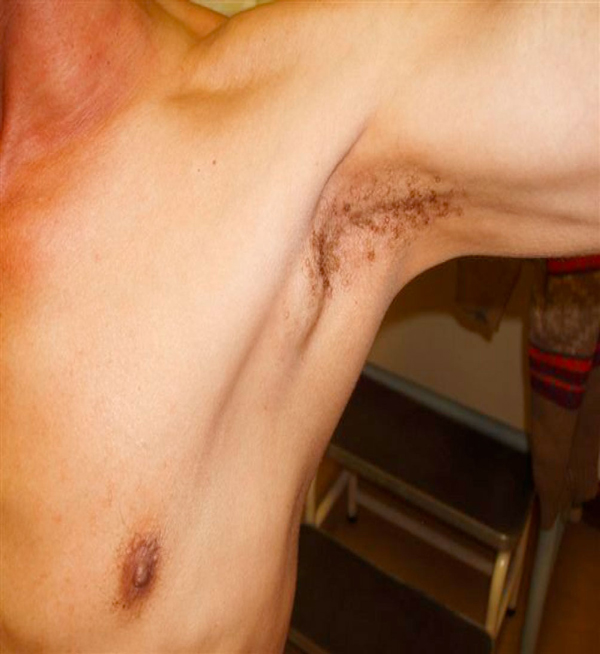
**Axillary node**.

On clinical examination, he was found to have an ulcerative lesion within the tonsillar fossa, involving both anterior and posterior tonsillar pillars. There was no extension into the base of the tongue and the lesion measured 5 cm maximum diameter. He had multiple ipsilateral cervical nodes involved, measuring from 0.5 cm to 3 cm in diameter. Levels 1, 2, 3, 4 and 5 were all involved (Figure 1). A single ipsilateral axillary node 6 cm by 5 cm was noted (Figure 2).

Clinical examination revealed no second primary tumour in the head and neck. Examination of the left upper limb, breast and chest wall was normal. His chest X-ray was clear. Computed tomography of the chest confirmed no evidence of another primary pathology.

On searching for distant metastases, examination of his abdomen and musculoskeletal system was normal. Liver function tests were normal, as were his serum calcium levels.

Histology from the oropharyngeal primary tumour confirmed a moderately differentiated SCC, and cytology from both cervical and axillary nodes was metastatic SCC, in keeping with the primary. He was diagnosed with T3N2bM1 oropharyngeal SCC.

The patient was offered a course of chemoradiotherapy but refused any treatment, and died 4 months after diagnosis.

## Discussion

The lymphatic drainage from the head and neck occurs through superficial and deep systems. The superficial group of nodes includes occipital, postauricular, parotid, facial, submandibular, submental and superficial cervical nodes, which lie with the external jugular vein. The deep group of nodes lies along the internal jugular vein. All head and neck lymphatics drain into the deep group, either directly or via the superficial group of nodes. The flow of the lymphatic system through the axilla is normally from the distal portions of the upper limb and the chest wall along the axillary vein toward the subclavian venous system [1].

In cases where axillary metastases have been reported in the literature, the mean time to axillary metastasis from successful locoregional control was 17 months, with a range of 3 to 40 months [2]. Previous reports suggest that axillary metastasis occurs because of altered lymphatic anatomy caused by previous treatment of the neck, and a subsequent recurrence or second primary may then seed down the new, aberrant lymphatic channels to the axilla [3]. It has also been suggested that complex and variable connections may exist between the cervical lymphatics and the axillary and/or chest lymphatics, with axillary metastases found in 2% to 9% of patients with head and neck cancer at autopsy [3].

In a previously untreated neck, the occurrence of axillary metastases is more difficult to explain. We postulate that the metastatic cancer cells made their way to the axillary node by one of two mechanisms. First, the cells could have traveled distally down the lymphatic system from the thoracic duct to the axillary lymphatics. Second, the fat in level 5 is continuous with the axillary fat, and spread via this pathway is also a possibility. Our patient did present with positive nodes in level 5, which would support this possible means of spread. It is possible that the tumor itself may induce alterations in normal drainage patterns [1].

Although axillary spread is rare, Koch [4] does suggest routine monitoring of the axillary nodes in patients who have developed recurrent or new primary disease in the head and neck after previous treatments of the neck with surgery and/or radiotherapy. Our case is a reminder to clinicians of the need to examine the axilla in patients with head and neck primaries, particularly those with level 5 nodal involvement at initial presentation.

## Conclusion

Involvement of axillary nodes in head and neck cancer is rare. This report presents a patient who had axillary nodal involvement at the time of his primary presentation with an oropharyngeal SCC. Previous reports in the literature suggest that the axilla may be involved in patients who have had previous neck surgery, as the result of altered lymphatic drainage. We suggest that the axilla be examined routinely in head and neck cancer, particularly when the level 5 nodes are involved.

## Consent

Written informed consent was obtained from the patient's family for publication of this case report and any accompanying images. A copy of the written consent is available for review by the Editor in Chief of this journal.

## Competing interests

The authors declare that they have no competing interests.

## Authors' contributions

JL was involved in the drafting of the manuscript and critically revising it for important intellectual content. BM managed the patient, obtaining all tissue samples and imaging. BM was the first author. JL read and approved the final manuscript.
